# Comparative Analysis of the In Vitro and In Vivo Antioxidant and Anti-Inflammatory Capacities of Lycopene Extracts from Different Sources

**DOI:** 10.3390/foods15101765

**Published:** 2026-05-17

**Authors:** Daolin Mou, Dajiang Ding, Lingyun Liu, Junning Pu, Guihua Xu, Jie Ren, Jing Lyu, Wanxue Wen

**Affiliations:** 1 School of Food and Liquor Engineering (School of Wuliangye Baijiu), Sichuan University of Science and Engineering, Yibin 644000, China; 2 State Key Laboratory of Neuroscience, Institute of Neuroscience, Center for Excellence in Brain Science and Intelligence Technology, Chinese Academy of Sciences, Shanghai 200031, China; 3 Key Laboratory for Animal Disease-Resistance Nutrition of China Ministry of Education, Institute of Animal Nutrition, Sichuan Agricultural University, Chengdu 611130, China

**Keywords:** lycopene, different sources, anti-oxidation, anti-inflammation, ultrasonic-assisted extraction

## Abstract

The sources of natural lycopene are diverse, and lycopene from different sources may have differences in functional characteristics and bioavailability. In this study, lycopene was extracted from tomatoes, cherry tomatoes, red guavas, carrots, and watermelons by ultrasonic-assisted extraction, and the structures were characterized. The differences in their in vitro and in vivo antioxidant capacities and anti-inflammatory capacity in vivo were compared. The results showed that under the extraction conditions of this experiment (sample: ethyl acetate: 1:5 m/v, 40 °C, 600 W, 40 kHz, 30 min), lycopene (primarily all-*trans* structure) from different sources could be effectively extracted from the above five raw materials. The concentration of lycopene extracted from the four samples except tomatoes (14.03 ± 1.08 mg/100 g fresh weight (FW)) was about 30 mg/100 g FW. The analysis of the in vitro antioxidant capacity of lycopene from five different sources showed that the 2,2′-azino-bis (3-ethylbenzthiazoline-6-sulfonic acid) (ABTS), diphenyl-1-picrylhydrazyl (DPPH) scavenging rates and ferric reducing antioxidant power (FRAP) concentration of the red guava lycopene-rich sample were significantly higher than those of the other four sources of lycopene. Based on the in vitro performance of lycopene from five sources, further in vivo experiments (using only the tomato and red guava groups) also found that compared with lycopene from tomatoes, lycopene-rich extract from red guavas could significantly increase the antioxidant enzyme activities and total antioxidant capacity in the serum, liver and gastrocnemius muscle (GAS) of mice; reduce the malondialdehyde (MDA) concentration; and also increase the expression of antioxidant-related genes (*GPx*, *CAT*, *SOD1*, etc.) in the liver and GAS of mice by regulating the Nrf-2/keap1 signaling pathway. In addition, mice in the guava-derived lycopene-rich group had lower serum levels of tumor necrosis factor-α (TNF-α) and interleukin-6 (IL-6). In summary, these results indicated that the lycopene-rich extract derived from red guava demonstrated higher antioxidant activity both in vitro and in vivo as well as enhanced anti-inflammatory capabilities within the body, providing an important reference for its application in the food industry and functional foods.

## 1. Introduction

Lycopene is a naturally occurring pigment found in plants, classified as a carotenoid with the chemical formula C_40_H_56_ and a molecular weight of 536.89 Da [1]. It possesses 13 unique double bonds, 11 of which are conjugated, accounting for its potent antioxidant properties [2]. In nature, lycopene exists predominantly in the stable all-*trans* configuration, which exhibits superior antioxidant capacity compared to its *cis*-isomers [3]. The presence of unsaturated double bonds renders lycopene susceptible to oxidative degradation or isomerization under environmental stressors such as light, oxygen, acid, and heat, leading to structural transformation from the native all-*trans* isomer to *cis*-isomers and consequently affecting its bioavailability [1]. Lycopene has demonstrated the ability to reduce reactive oxygen species (ROS) and quench singlet oxygen, nitrogen dioxide, hydroxyl radicals, and hydrogen peroxide [4]. Its singlet oxygen quenching capacity is twice that of β-carotene and ten times that of α-tocopherol [2]. In vivo, lycopene also enhanced the activity of antioxidant enzymes such as catalase (CAT), superoxide dismutase (SOD) and glutathione peroxidase (GSH-Px) [5,6,7]. Owing to its robust antioxidant efficacy, lycopene exhibited significant beneficial effects against inflammation, cardiovascular diseases, and prostate cancer [3]. In addition, both in vitro and in vivo studies have shown that lycopene possesses anti-inflammatory properties, particularly in modulating intestinal inflammation [7,8,9]. Consequently, lycopene has been widely applied in health foods, pharmaceuticals, cosmetics, and agriculture and is recognized as a Class A nutrient by the Food and Agriculture Organization of the United Nations, Food Additives Committee and the World Health Organization [10]. It is primarily found in the ripe fruits of plants such as tomatoes, watermelons, guavas, carrots, pumpkins, and papayas. Although tomatoes represent the most economical natural reservoir and currently serve as the primary source for lycopene production, other fruits and vegetables such as guavas, watermelons, and carrots also offer abundant resources and renewability, holding potential applications of significant value to industries such as food processing [1,11].

The primary methods for extracting natural lycopene include traditional solvent extraction, supercritical fluid extraction (SFE), enzyme-assisted extraction (EAE), microwave-assisted extraction (MAE), and ultrasonic-assisted extraction (UAE) [1]. Traditional solvent extraction is time-consuming, solvent-intensive, and inefficient, with the added risk of thermal denaturation of biomolecules due to high temperatures [3]. SFE extraction is considered a safe and straightforward process, but it involves high operational and equipment costs [12]. Enzymatic hydrolysis breaks down lycopene-rich cellular structures through enzymatic action to release lycopene, offering a gentle process, but it has relatively high enzyme costs. MAE is a simple, effective, and rapid method that utilizes microwaves to rapidly heat polar components within cells, promoting the efficient release of lycopene into the extraction solvent. UAE is a highly efficient non-thermal process that reduces the extraction time and solvent volume while preserving compound bioactivity. UAE employs ultrasound to disrupt cell walls, releasing lycopene into the extraction solvent [13,14]. Considering the advantages and disadvantages of these extraction methods, and based on the principles of extracting food-grade lycopene, environmental sustainability, and minimizing extraction time and energy consumption, we selected the UAE method for extracting lycopene from various plant sources. For the extraction solvent, we chose ethyl acetate, a food-grade solvent classified as GRAS (generally regarded as safe) by the Food and Drug Administration (FDA) [15,16].

Li et al. (2025) found that the lycopene content varies among different tomato cultivars, leading to differences in their antioxidant capacity [17]. Similarly, the diverse sources of natural lycopene result in variations in its content, bioavailability, and stability. Furthermore, lycopene from different sources may exhibit significant differences in antioxidant activity both in vivo and in vitro. To date, no studies have systematically analyzed and compared the differences in the in vivo and in vitro antioxidant and anti-inflammatory capabilities of lycopene from different sources. Therefore, investigating the extraction and functional characteristics of lycopene-rich extracts from different sources holds significant importance. This study employed ethyl acetate as a solvent and UAE to isolate lycopene from five distinct plant sources. By comparing the in vivo and in vitro antioxidant and anti-inflammatory capacities of lycopene from these sources, it aims to provide reference for optimizing raw material selection in industries such as food processing and for advancing functional food innovation.

## 2. Materials and Methods

### 2.1. Ultrasound-Assisted Extraction of Lycopene

Fresh tomatoes (*Solanum lycopersicon*, cv. *Provence*, origin: Chengdu, Sichuan Province), cherry tomatoes (*Solanum lycopersicum* var. *cerasiforme*, origin: Chengdu, Sichuan Province), red guava (*Psidium guajava* L., origin: Yulin, Guangxi Province), watermelon (*Citrullus Lanatus*, origin: Yibin, Sichuan Province) and carrots (*Daucus carota* var. *sativa Hoffm*., origin: Panzhihua, Sichuan) were purchased from a local supermarket in Yibin (28°45′07″ N, 104°38′35″ E), Sichuan, in April 2025. The samples were washed thoroughly with clean water. The skin and seeds were removed, and only the flesh was retained.

The samples were cut into small pieces, processed in a blender (Philips, HR2062, Zhuhai, China) and homogenized for 5 min to form a puree. The ultrasonic-assisted extraction protocol was modified from Mozafari, Marinaccio and Kuvendziev [13,18,19]. About 5 g of homogenized sample was placed into a 50 mL centrifuge tube (Corning, Corning, NY, USA), and 25 mL of ethyl acetate (HPLC grade, Shanghai Macklin Biochemical Co., Ltd., Shanghai, China) was added. The mixture was vortexed for 5 min and then sonicated at 600 W, 40 kHz and 40 °C for 30 min using a rectangular ultrasonic processor (F-100S, Shenzhen Yujie Cleaning Equipment Co., Ltd., Shenzhen, China, 500 × 300 × 200 mm internal dimensions). After sonication, the mixture was vortexed again for 5 min and then centrifuged at 4400 rpm for 5 min at room temperature. The supernatant was collected and filtered through a 0.45 μm hydrophobic PTFE membrane needle-type filter (Whatman, Kent, UK) for subsequent analysis. The extraction process was conducted in the absence of light throughout. Each sample was replicated three times.

### 2.2. Moisture and Scanning Electron Microscope Analysis

The samples’ moisture content was measured according to Tan et al. (2021) [20]. Approximately 5 g of the sample was placed in a 105 °C oven to dry until a constant weight was achieved. The results of moisture analysis are shown in Supplementary Figure S1.

The surface morphology and form of the two different samples were examined utilizing SEM (Sigma 360, Carl Zeiss AG, Oberkochen, Germany) under high vacuum at an accelerating voltage of 5 kV for further investigation [14,21]. The two different samples were a dried sample (dried samples used for moisture content determination) and a liquid sample (liquid samples after lycopene extraction with ethyl acetate). Before the SEM images were taken, if samples were needed, they were coated with conductive material (Au). The SEM images of the samples were taken at two different magnifications: 20k× and 10k×.

### 2.3. Lycopene Content and Percentage Recovery Determination

The determination of the lycopene concentration was carried out according to the method described by Kuvendziev and Zheng with some modifications [13,22]. The absorbance of the lycopene-rich extract solution (liquid sample) was measured using a UV–Vis spectrophotometer (UV-1900i, Shimadzu, Tokyo, Japan). A quartz cuvette with a path length of 10 mm and Δλ = 1 nm were used. The baseline was automatically adjusted using ethyl acetate as the blank, and the analytical spectra within the range of 300–600 nm were recorded. In this experiment, the lycopene exhibited three distinct peaks at wavelengths of 447 nm, 473 nm and 505 nm. To minimize the interference from other carotenoids, the peak obtained at 505 nm was used for further analysis [13,21,22]. The standard curve was prepared as follows: the lycopene standard (UV ≥ 98%, Beijing Solarbio Science & Technology Co., Ltd., Beijing, China) was dissolved in ethyl acetate to prepare a stock solution and diluted to working solutions of various concentrations. The absorbance was measured at 505 nm using a UV–Vis spectrophotometer, and the absorbance–concentration standard curve (*y* = 14.136*x* − 0.7092, *R*^2^ = 0.9996) was established. The content of lycopene in the samples was calculated using the standard curve, and the results are expressed as milligrams of lycopene per 100 g of pulp (mg lycopene 100 g^−1^ FW).

The percentage recovery (percentage yield, Y%) was determined based on previous studies [2,23]. The calculation formula is as follows:

(1) $$Y\%=\frac{Amount\;of\;lycopene\;extracted\;in\;a\;single\;extraction\;step\;(mg/100\;g)}{Maximum\;initial\;mass\;of\;lycopene\;in\;the\;sample\;(mg/100\;g)}\times 100$$

### 2.4. HPLC Analysis for Lycopene

The extracted lycopene (liquid sample) was identified and re-quantified using a high-performance liquid chromatography–diode array detector (HPLC-DAD) instrument (Agilent 1260, Santa Clara, CA, USA) equipped with a reversed phase InfinityLab Poroshell 120 EC-C18 (150 mm × 4.6 mm × 4 μm, Santa Clara, CA, USA) and DAD. The chromatographic conditions were a combination of previous studies, with some modifications [2,16,22,23,24,25]. The separation method consisted of a gradient mode with a mobile phase composed of two solvents at a flow rate of 1.5 mL/min: methanol (HPLC ≥ 99.9%, Innochem, Beijing, China)/acetonitrile (ACS Reagent, for HPLC, Honeywell, Honeywell, NC, USA) (95:5) (solvent A) and water (solvent B). The mobile phase stepwise gradient program was as follows: 0–4 min: 97% A, 3% B; 5–29 min: 100% A, 0% B; and 30 min onwards: 100% A, 0% B. The column temperature was 30 °C, and the injection volume was 10 μL. The detection wavelength was 473 nm. All-*trans*-lycopene in the sample was identified based on the retention time and the spectrum as compared to the standard; the *cis*-isomers were estimated according to the method described by Yi and Ho; and the β-carotene was estimated according to the method described by Mozafari [19,26,27]. The lycopene standard (HPLC ≥ 98%, Beijing Solarbio Science & Technology Co., Ltd., Beijing, China) was also dissolved in ethyl acetate (HPLC grade, Shanghai Macklin Biochemical Co., Ltd., Shanghai, China) for preparing a stock solution, and then the stock solution was diluted to 25 mg/mL, 20 mg/mL, 15 mg/mL, 10 mg/mL and 5 mg/mL to establish a standard curve (*y* = 15.8012*x* − 8.4881, *R*^2^ = 0.99909). The lycopene concentration was calculated using the standard curve. The percentage purity of the extracted lycopene was calculated by the area normalization method.

### 2.5. FTIR Analysis

The ATR-FTIR was conducted in the same way as that described by Mou [7] and was carried out using an IRAffinity-1S spectrometer (Shimadzu Corporation, Tokyo, Japan). The samples (liquid samples) were tested at a resolution of 2 cm^−1^ with 32 scans over the range between 400 and 4000 cm^−1^.

### 2.6. Determination of Antioxidant Capacity of Lycopene

To compare the differences in antioxidant capacity of lycopene from different sources, all samples were diluted to the lycopene concentration of 20 mg/L based on the lycopene concentration results. The 2,2′-azino-bis(3-ethylbenzthiazoline-6-sulfonic acid) (ABTS) radical scavenging ability, diphenyl-1-picrylhydrazyl (DPPH) radical scavenging ability and ferric reducing antioxidant power (FRAP) of samples were respectively determined using the corresponding kits (Beijing Solarbio Science & Technology Co., Ltd., Beijing, China) in accordance with the instructions.

### 2.7. Analysis of the Antioxidant Capacity of Lycopene In Vivo

The antioxidant capacity of lycopene may differ between in vivo and in vitro conditions. Evaluating its in vivo antioxidant capacity is crucial for assessing lycopene as a functional food ingredient. To further compare the differences in antioxidant capacity between lycopene-rich extracts from different sources in vivo, we conducted in vivo experiments based on our in vitro antioxidant analysis. Since the in vitro analysis primarily revealed higher antioxidant capacity in red guava-derived lycopene-rich samples, and tomatoes are the primary industrial source of lycopene, our in vivo experiments included two treatment groups: red guava-derived and tomato-derived lycopene extracts.

The in vivo experimental design refers to the previous research designed by Mou [7], and the trial was approved by the Animal Care and Use Committee of Sichuan Agricultural University (license number: CD-SYXK-2017-015). Thirty-six 4-week-old male mice (Balb/c mice, purchased from GemPharmatech Co., Ltd., Chengdu, China) were randomly divided into three treatment groups: control group (without added lycopene, CON, n = 12), the tomato-derived lycopene group (Tomato group, n = 12) and the red guava-derived lycopene group (Guava group, n = 12). The lycopene derived from tomatoes and red guavas was extracted using the aforementioned method. Subsequently, it was subjected to vacuum concentration using a rotary evaporator (RE-52AA, Shanghai Yarong Biochemical Instrument Factory, Shanghai, China) and then dried by freeze-drying (SCIENTZ-10ND, NingBo Scientz Biotechnology Co., Ltd., Ningbo, China) to obtain a solid powder form of lycopene. The lycopene powder obtained from different sources was added to mouse feed to formulate the corresponding treatment groups. The feed was kept away from light and high temperatures during preparation and storage. The basic diet fully meets the nutritional standards recommended by AIN-93. Based on our previous studies, the lycopene additive dosage is 300 mg/kg of feed [7]. The experiment lasted for 6 weeks. All mice were housed individually in cages, maintained at 22 ± 2 °C, and exposed to a 12 h light–dark cycle. Mice had free access to water and food. The body weight, average daily feed intake and food average daily gain of mice are shown in Supplementary Figure S2.

### 2.8. Analysis of Redox-Related Indicators in Mice

At the end of the in vivo experiment, mouse serum, liver and gastrocnemius muscles were collected for the following analyses. Catalase (CAT, Cat. No. A007-1-1), total superoxide dismutase (T-SOD, Cat. No. A001-1-2), glutathione peroxidase (GSH-Px, Cat. No. A005-1-2), total antioxidant capacity (T-AOC, Cat. No. A015-2-1), and malondialdehyde (MDA, Cat. No. A003-1-1) in mouse serum, liver and gastrocnemius muscles were analyzed using the corresponding commercial kits in accordance with the instructions (Nanjing Jiancheng Bioengineering Institute, Nanjing, China).

### 2.9. Analysis of Inflammatory Factors in Mouse Serum and Immunohistochemical Analysis of the Liver

Serum tumor necrosis factor-α (TNF-α, Cat. No. ml002095), interleukin 6 (IL-6, Cat. No. ml063159), interleukin-1β (IL-1β, Cat. No. ml106733) and interleukin 10 (IL-10, Cat. No. ml037873) concentrations were measured by using the commercial ELISA kits in accordance with the instructions (Shanghai Enzyme-linked Biotechnology Co., Ltd., Shanghai, China).

Immunohistochemistry of liver NF-κB p65, TNF-α and IL-6 were analyzed as in our previous research described by Mou [7]. Briefly, liver samples (n = 4) were collected, preserved in 4% paraformaldehyde solution (Beyotime, Shanghai, China), embedded in paraffin, and sectioned. After dewaxing, the sections were washed with a series of alcohol solutions, rehydrated with phosphate-buffered saline, boiled in citrate buffer (pH 6.0, 10 mmol·L^−1^), and then blocked with 3% bovine serum albumin for 30 min. Subsequently, the sections were incubated overnight at 4 °C with rabbit anti-NF-κB p65 (D14E12) antibody (1:500, #8242, Cell Signaling Technology, Danvers, MA, USA), mouse anti-TNF-α (1:50, #CL488-60291, Proteintech, Wuhan, China) and anti-IL-6 monoclonal antibody (1:100, ab290735, Abcam, Cambridge, UK). The sections were then incubated with specific secondary antibodies and 3,3′-diaminobenzothiazole tetrahydrochloride (DAB) to visualize the immune complexes. Images were captured using a BA200 digital microscope (Motic, Xiamen, China). Quantitative analysis was performed using ImageJ software (version 1.46, NIH, Bethesda, MD, USA).

### 2.10. Quantitative Analysis of Antioxidant-Related and Inflammation-Related Genes in Mice

The real-time quantitative PCR for antioxidant-related genes in the liver and gastrocnemius muscles of mice (n = 6) and liver inflammation-related genes was carried out according to the previous method described by Mou [7]. Briefly, total RNAs were extracted using the reagent (E.Z.N.A.^®^ Total RNA Kit I reagent, Omega Bio-Tek, Norcros, GA, USA). Then, the extracted RNA was reverse transcribed to cDNA using the reverse transcription reagent (HiScript^®^ III RT SuperMix for qPCR (+gDNA wiper) kit, Vazyme, Nanjing, China). Finally, fluorescence quantitative analysis was conducted using the quantitative kit (ChamQ Universal SYBR qPCR Master Mix kit, Vazyme, Nanjing, China). All procedures were carried out in accordance with the instructions of the kit. All the primers for antioxidant-related genes are listed in Table S1 of the Supplementary Materials.

### 2.11. Statistical Analyses

SPSS 26.0 software (SPSS Inc., Chicago, IL, USA) was used for statistical analysis. The data were analyzed using the one-way ANOVA procedure with Duncan’s multiple range tests to determine differences between each group. The data were tested for normality and homogeneity of variances (Shapiro–Wilk and Levene tests, respectively) before analysis. The data are presented as the mean ± standard error of the mean (SEM). Lycopene extraction experiments (the in vitro experiments) were performed with three independent biological replicates (n = 3). *p* < 0.05 indicates a significant difference between groups. The results were plotted with Origin 2021 (OriginLab, Northampton, MA, USA) or GraphPad Prism 9.5 software (Graphpad software, San Diego, GA, USA).

## 3. Results and Discussion

### 3.1. Lycopene Extraction and Its Characteristics

**Figure 1:**
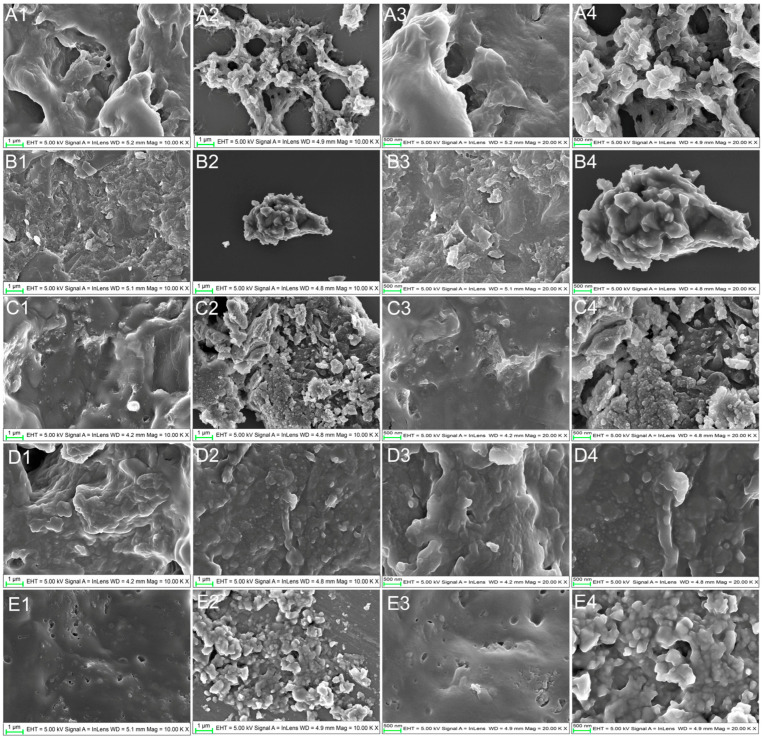
SEM images of different samples of lycopene extraction. (**A1**–**A4**) Tomato; (**B1**–**B4**) cherry tomato; (**C1**–**C4**) red guava; (**D1**–**D4**) carrot; (**E1**–**E4**) watermelon. (**A1**–**E1**, **A3**–**E3**) Pre-extraction samples; (**A2**–**E2**, **A4**–**E4**) post-extraction samples. (**A1**–**E1**, **A2**–**E2**) 10k×; (**A3**–**E3**, **A4**–**E4**) 20k×.

**Figure 2:**
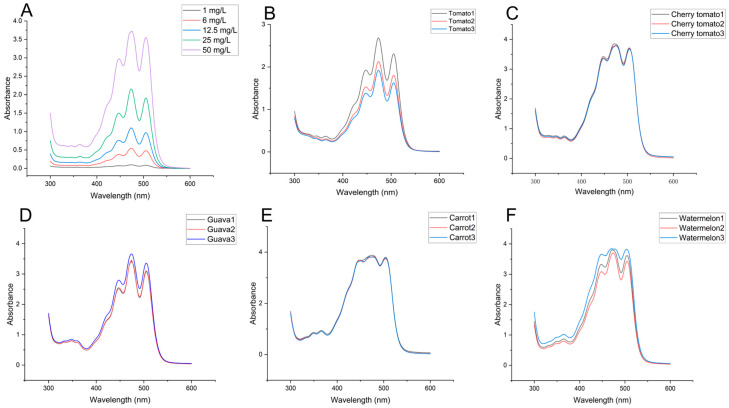
UV–Vis spectra of lycopene from different sources. (**A**) Lycopene reference standard; (**B**) tomato; (**C**) cherry tomato; (**D**) red guava; (**E**) carrot; (**F**) watermelon.

**Figure 3:**
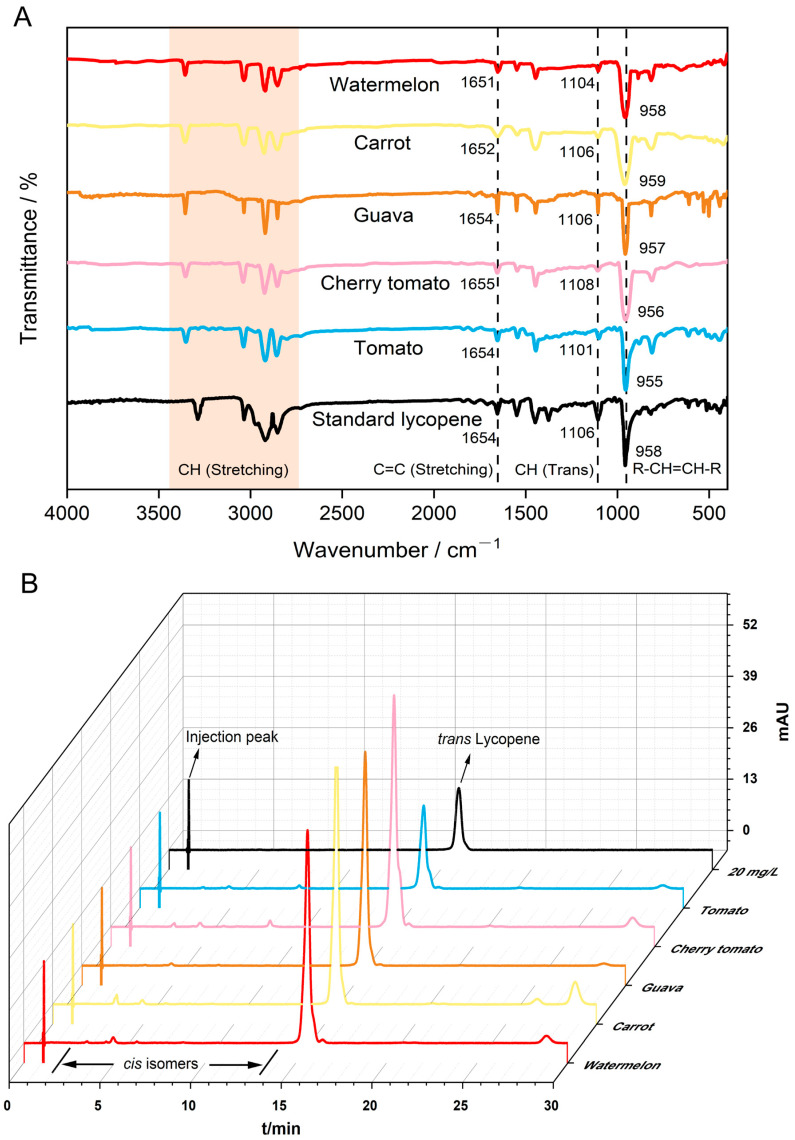
HPLC chromatogram and FTIR spectra of lycopene extracted from different sources. (**A**) FTIR spectrum analysis; (**B**) HPLC chromatogram.

Under the extraction conditions employed in this experiment (sample: ethyl acetate: 1:5 m/v, 600 W, 40 °C, ultrasonic-assisted extraction for 30 min), SEM analysis revealed structural differences between pre- and post-extraction samples. The post-extraction samples exhibited disrupted tissue cell structures (Figure 1), facilitating improved lycopene dissolution into the ethyl acetate solvent. Previous studies had also demonstrated that ultrasonic treatment disrupts plant cell walls, facilitating lycopene extraction [14]. Lycopene exhibited three characteristic absorption peaks in both n-hexane solutions (445, 472, and 502 nm) and acetone–petroleum ether solutions (446, 472, and 504 nm) in UV–Vis spectroscopy [13,22]. Similarly, this study found that both lycopene standards dissolved in ethyl acetate, and the lycopene-rich extracts from five samples exhibited three characteristic peaks (447 nm, 473 nm, and 505 nm) within the 300–600 nm wavelength range (Figure 2). However, differences in peak shapes and peak overlap were observed among the lycopene-rich extracts from different samples (Figure 2). These variations stemmed from differing lycopene contents in replicate experiments and the presence of distinct isomers in extracts from various sources. Therefore, to further characterize the lycopene from the five different sources, FTIR analysis was conducted on the samples (Figure 3A). FTIR spectral analysis of the lycopene standard and the five extracted samples revealed four absorption peaks within the 2850–3360 cm^−1^ range, corresponding to C–H stretching vibrations [7,28]. All samples exhibited absorption peaks near 1654 cm^−1^ and 1549 cm^−1^ corresponding to C=C stretching vibrations [7,28]. Additionally, the lycopene standard exhibited two infrared absorption peaks at 1448 cm^−1^ and 1375 cm^−1^, while the five lycopene-rich extract samples showed a single peak near 1446 cm^−1^. All these peaks correspond to C-H bond bending vibrations. The absorption peak near 1106 cm^−1^ in all samples corresponds to CH (trans) [28]. A strong characteristic lycopene absorption peak appeared near 958 cm^−1^ in all samples, corresponding to the deformation vibration of R–CH=CH–R in lycopene [7,28,29,30].

The lycopene structure in the extracts was further confirmed by HPLC-DAD, while the purity of all-*trans* lycopene in the extracts was determined. The degradation and isomerization of the extracted samples were also analyzed (Figure 3B and Table 1). The peaks corresponding to *trans*-lycopene in the extracts were identified by comparison with the standard. Other carotenoid types and *cis*-isomers were estimated based on previous studies [26,31,32]. As shown in Figure 3B, the peak at approximately 15.8 min retention time corresponds to all-*trans* lycopene. Three *cis*-isomer peaks were detected near 3.5, 4.9, and 8.8 min elution times, while a β-carotene peak appeared around 28.8 min. The content of all-*trans* lycopene and other carotenoids varied among lycopene-rich extracts from different sources. As shown in Table 1, cherry tomatoes and carrots exhibited higher levels of all-*trans* lycopene than the other three sources, while guavas had the lowest levels of *cis*-lycopene and β-carotene; carrots contained significantly higher levels of β-carotene than the other four sources. These results indicate that under the current extraction conditions, lycopene can be effectively extracted from five samples: tomatoes, cherry tomatoes, red guavas, carrots, and watermelons.

### 3.2. Lycopene Concentration and Percentage Recovery in Extracted Samples

**Figure 4:**
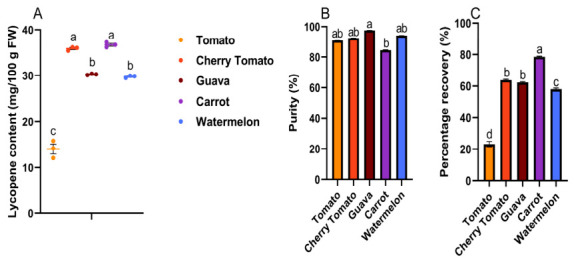
Lycopene concentration and purity in different extracted samples. (**A**) Lycopene concentration in extracts from different sources (n = 3 per group); (**B**) lycopene purity in different extracted samples; (**C**) percentage recovery of different extracted samples. Data are expressed as means ± SEM. ^a–d^ Columns with different letters indicate significant differences (*p* < 0.05).

In this experiment, the lycopene content of the five sources was found to be essentially the same when measured using UV–Vis (Figure 4A) and HPLC-DAD (Table 1) methods. As shown in Figure 4A, the lycopene concentrations extracted from cherry tomatoes (35.89 ± 0.23 mg/100 g FW) and carrots (36.74 ± 0.33 mg/100 g FW) were significantly higher than those from the other three types, while guavas (30.26 ± 0.08 mg/100 g FW) and watermelons (29.76 ± 0.14 mg/100 g FW) exhibited significantly higher lycopene concentrations than tomatoes (14.03 ± 1.08 mg/100 g FW). This funding suggested that there were significant differences in the lycopene content among lycopene-rich extracts from different sources. Using conventional extraction methods, the lycopene content extracted from watermelon pulp was 8.20–59.17 mg/100 g, the lycopene extracted from red guava byproducts was 258.05 ± 1.38 μg/100 g wet decanter, while tomato pulp yielded 2.01 ± 0.09 mg/100 g FW and carrots yielded 50.82 ± 0.18 μg/g [24,33,34,35]. Therefore, under the extraction conditions of this experiment, the lycopene concentration obtained was higher than that achieved by traditional methods. This is because the UAE method could effectively disrupt plant cell walls and facilitate the release of more lycopene into the extraction solvent. Previous studies have reported that the lycopene contents extracted from tomato paste waste, cherry tomatoes and tomato peel by UAE reached 104.85 mg/100 g, 168.2 mg/g and 146.6 mg/100 g, respectively [36,37]. Additionally, the UAE extraction of lycopene from tomato pomace yielded approximately 200 mg/kg after a single extraction and around 600 mg/kg after three extractions [13]. Compared to the lycopene concentrations obtained using UAE in the above studies, a single extraction cycle yielded higher lycopene concentrations than traditional methods, suggesting that UAE effectively enhances the lycopene extraction efficiency.

As shown in Figure 4B, HPLC analysis revealed that the lycopene-rich extract from red guava exhibited the highest purity (98%). Tomatoes, cherry tomatoes, and watermelon followed with purities exceeding 90%, while carrot-derived lycopene-rich extract had the lowest purity at 85%. Studies have found that when UAE was used to extract lycopene from tomatoes, the purity of all-*trans* lycopene was 96.81%, while the purity of lycopene extracted from red grapefruit was 91.5% [38,39]. The purity of the extracted lycopene varies depending on the extraction method and source. Under the extraction conditions used in this study, it demonstrated a higher all-*trans* lycopene purity compared to pomegranates from other sources, indicating its excellent application prospects.

As shown in Figure 4C, carrots exhibited the highest lycopene-rich extraction yield, while tomatoes had the lowest. Cherry tomatoes and red guavas both had extraction yields of around 60%. Using a lecithin-based olive oil microemulsion to extract lycopene, it was found that the extraction yield was approximately 63% after one extraction cycle, while the yield was approximately 88% after four cycles [40]. Our data indicate that tomato yielded a low extraction yield, while the other four sources all yielded high extraction yields in a single extraction.

### 3.3. In Vitro Antioxidant Activity Analysis of Lycopene from Different Sources

**Figure 5:**
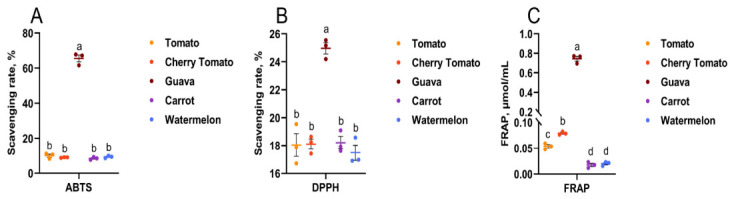
Lycopene ABTS and DPPH scavenging rates and FRAP concentration in different extracted samples. (**A**) ABTS scavenging rate. n = 3 per group. (**B**) DPPH scavenging rate. n = 3 per group. (**C**) FRAP concentration. n = 3 per group. Data are expressed as means ± SEM. ^a–d^ Columns with different letters indicate significant differences (*p* < 0.05).

The antioxidant capacity of lycopene is a key functional characteristic. While studies have identified variations in the antioxidant activity among lycopene extracted via different methods, lycopene isomers, and tomato cultivars, whether lycopene from different extraction sources exhibits differences in antioxidant capacity remains to be further investigated [10,17,31,41,42]. To verify the differences in antioxidant capacity among lycopene sources in this study, we analyzed and compared five lycopene sources using ABTS, DPPH, and FRAP assays (Figure 5). To eliminate variations caused by differing lycopene concentrations, all samples were diluted to uniform lycopene concentrations prior to antioxidant parameter measurements. The results revealed that the red guava-derived lycopene-rich extract exhibited significantly higher ABTS and DPPH scavenging rates and FRAP contents than the other four sources (Figure 5A,B). As shown in Figure 5C, the cherry tomato lycopene-rich extract demonstrated higher FRAP contents than the lycopene-rich extracts from tomatoes, carrots, and watermelons, while the tomato lycopene-rich extract exhibited a higher FRAP content than carrot and watermelon lycopene. These results suggested that the red guava-derived lycopene-rich extract showed the highest antioxidant ability among them.

It is known that guava is a fruit that is rich in antioxidant active ingredients such as polyphenols, anthocyanins, flavonoids, triterpenoids, ascorbic acid and carotenoids, and it is popularly used for food and medicine purposes. A previous study on organic solvent extraction indicated that the two types of carotenoids with the highest content in the extract of pink guava are lycopene and β-carotene [33]. In the present study, the red guava-derived lycopene-rich extract exhibited the highest purity (more than 98%), while the carrot-derived lycopene-rich extract had the lowest purity. Consequently, at equivalent lycopene concentrations in the extracts, the carrot extract contained higher levels of other carotenoids. By contrast, the red guava extract contained only 0.48 mg/100 g FW of other carotenoids in this study. A previous study reported that lycopene has a singlet oxygen quenching capacity twice that of β-carotene [2].

Under the experimental conditions of this study, lycopene-rich extract from red guava exhibited an ABTS scavenging rate three times higher and a significantly higher FRAP concentration (approximately seven times higher) than those from the other four sources. These results indicated that the difference in the purity of all-*trans* lycopene was the main factor responsible for the variations in antioxidant capacity of lycopene-rich extracts from different sources. Furthermore, the lycopene in fresh tomatoes exists as all-*trans*-isomers and can be converted into *cis* isomers under the external or internal environment. The conversion from the all-*trans* configuration to the *cis* isomer needs relatively high energy, so the lycopene exists predominantly in the stable all-*trans* configuration in nature. Moreover the all-*trans* structure exhibits superior antioxidant capacity compared to its *cis*-isomers [3]. Within the context of the present study, the higher antioxidant capacity observed in the red guava-derived lycopene-rich extracts might be associated with *trans*-lycopene [43]. In summary, these results suggested that with respect to the variations in the in vitro antioxidant capacity of lycopene from different sources, the lycopene-rich extract from red guava showed higher antioxidant activity among them. However, in the in vitro experiments of this study, the lycopene samples were not pure compounds but lycopene-rich extracts. These extracts might contain other interfering factors, such as co-extracted compounds or matrix effects. Accordingly, significant differences in the in vitro antioxidant capacity were observed among lycopene-rich extracts from different botanical sources, and the mechanistic interpretation warrants further investigation.

### 3.4. In Vivo Antioxidant Capacity Analysis of Lycopene

The analysis results of the in vitro antioxidant capacity of lycopene showed that there were differences in the antioxidant capacity of lycopene-rich extracts from different sources. In particular, the lycopene-rich extracts from red guava had higher ABTS, DPPH and FRAP values than those from other sources. Based on the results of the in vitro antioxidant capacity test, a further in vivo experiment was performed to explore whether red guava-derived lycopene-rich extract possessed superior antioxidant capacity. It is well known that lycopene contains unsaturated double bonds, making it highly susceptible to isomerization under environmental stressors such as light and heat. It is converted from the natural all-*trans* isomer to cis isomers, thereby affecting its bioavailability. Additionally, the bioavailability of carotenoids is also related to their types, molecular structures, intake patterns, food matrices, genetic factors, and dietary interactions. Therefore, in the present study, we focused on investigating the effects of lycopene-rich extracts from different sources on the in vivo antioxidant capacity under identical experimental conditions.

**Figure 6:**
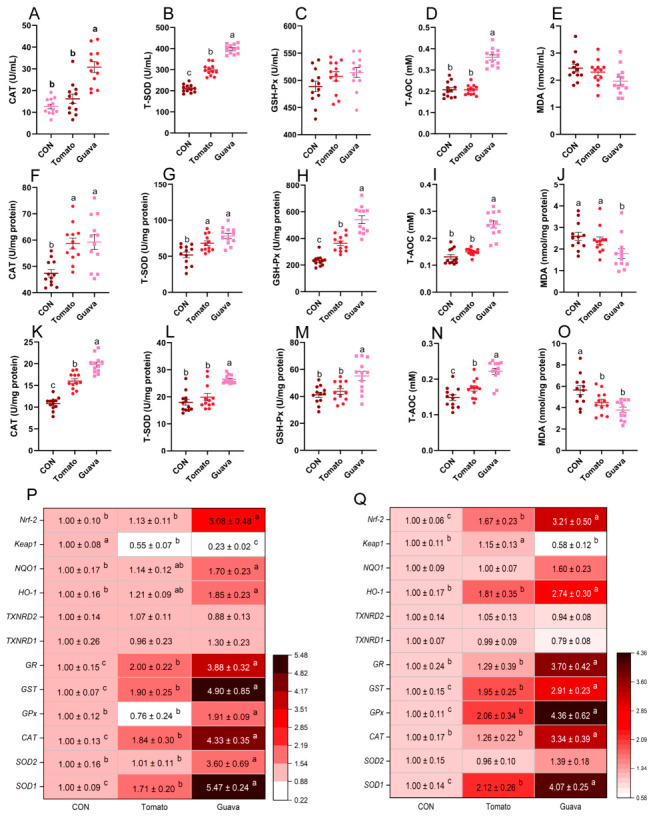
Effect of different sources of lycopene on antioxidant capacity of mice. (**A**–**E**) Redox status-related indicators in mouse serum (n = 12 per group). (**F**–**J**) Redox status-related indicators in mouse liver (n = 12 per group). (**K**–**O**) Redox status-related indicators in mouse GAS (n = 12 per group). (**P**) Relative mRNA expression of mouse liver redox-related genes detected by RT-PCR (n = 6 per group). (**Q**) Relative mRNA expression of mouse GAS redox-related genes detected by RT-PCR (n = 6 per group). Data are expressed as means ± SEM. ^a–c^ Columns with different superscript letters indicate significant differences (*p* < 0.05).

In the in vivo experiments of this study, the lycopene-rich extracts were vacuum-concentrated using a rotary evaporator and then freeze-dried with a freeze dryer to finally obtain purified lycopene. The method of lycopene purification from red guava and tomato was developed by Vasconcelos [44]. As shown in Figure 6A–E, compared with the control group and the tomato-derived lycopene-rich group, mice in the red guava-derived lycopene-rich group had higher serum CAT and T-SOD activities, as well as higher T-AOC concentrations. As shown in Figure 6F–O the red guava-derived lycopene-rich group exhibited higher CAT, T-SOD, and GSH-Px enzyme activities, as well as higher T-AOC concentrations in mouse liver and GAS compared to the tomato-derived lycopene-rich group, while the MDA concentrations were lower. Further analysis of antioxidant-related gene expression in mouse liver and muscle revealed that the red guava-derived lycopene-rich group had significantly increased *Nrf-2*, *HO-1*, *GR*, *GST*, *GPx*, *CAT*, *SOD1*, and *SOD2* expression and decreased *Keap1* expression in mouse liver (Figure 6P). Similarly, in the red guava-derived lycopene-rich group, the expression levels of the *Nrf-2*, *NQO1*, *GR*, *GST*, *GPx*, *CAT*, and *SOD1* genes in the GAS were significantly higher than those in the tomato-derived lycopene-rich group, and *Keap1* gene expression levels were lower (Figure 6Q). These findings showed that both the red guava-derived lycopene-rich and tomato-derived lycopene-rich groups exhibited strong in vivo antioxidant capacity, and the antioxidant activity of the red guava-sourced lycopene-rich extract was significantly higher than that of the tomato-sourced lycopene-rich extract, which was consistent with the results of the in vitro study. The possible reason was that the red guava-derived lycopene-rich extract had a higher purity of all-*trans* lycopene, which contributed to its higher bioavailability. Moreover, lycopene could enhance the expression of antioxidant-related enzymes (CAT, SOD, Gpx) through the Nrf-2/Keap1 signaling pathway [5,7,45]. Vasconcelos et al. (2017) found that guava-derived lycopene reduced inflammation levels in mice and increased GSH levels [44]. Lycopene-rich extract from red guava (*Psidium guajava* L.) could also decrease plasma triglycerides and improve oxidative stress biomarkers [46]. The results of this experiment indicated that the guava-derived lycopene-rich extract significantly the increased antioxidant capacity in mouse serum, liver, and GAS by regulating the Nrf-2/Keap1 pathway-mediated expression of antioxidant-related genes. However, the lycopene-rich extracts extracted and purified from red guava and tomato were not pure compounds, and the underlying mechanism remains to be further investigated.

### 3.5. Effects of Lycopene from Different Sources on Inflammatory Status in Mice

**Figure 7:**
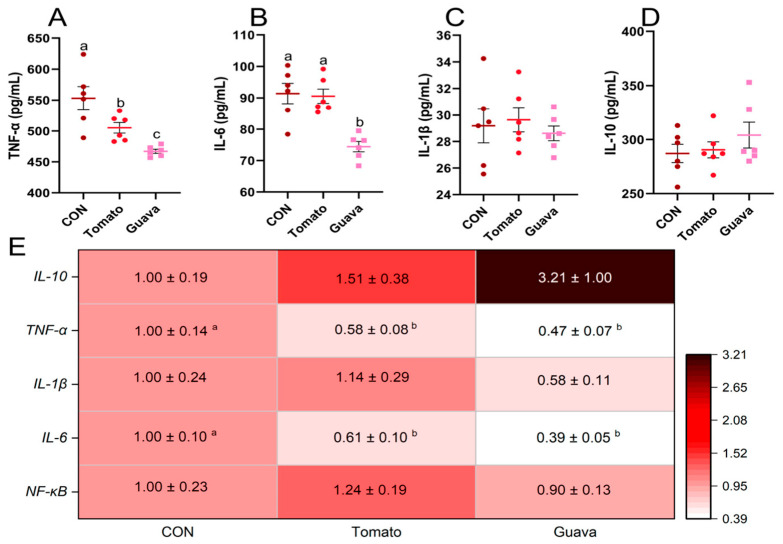

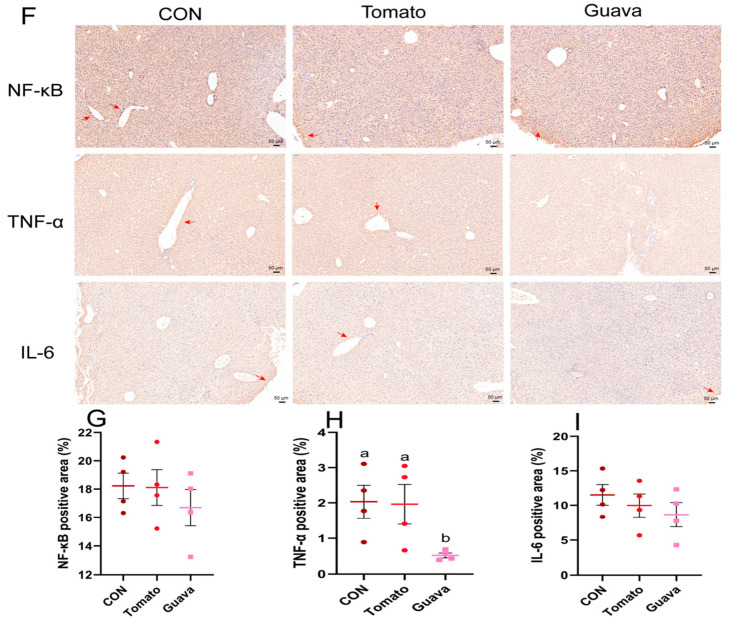
Effect of different sources of lycopene on inflammation-related factors in mice. (**A**–**D**) Levels of inflammasome-related factors in mouse serum (n = 6 per group). (**E**) Relative mRNA expression of mouse liver inflammation-related genes detected by RT-PCR (n = 6 per group). (**F**–**I**) IHC staining of NF-κB, TNF-α and IL-6 in mouse liver (n = 4 per group). Scale bar, 50 μm. The red arrows indicate typical areas of positive cells. Data are expressed as means ± SEM. ^a–c^ Columns with different superscript letters indicate significant differences (*p* < 0.05).

As shown in Figure 7, serum TNF-α and IL-6 concentrations in mice in the guava-derived lycopene-rich group were significantly lower than those in the other two groups. Further analysis of liver inflammation-related gene expression also revealed that *TNF-α* and *IL-6* gene expression levels were lower in the tomato- and guava-derived lycopene-rich groups than in the control group. There were no significant changes in the mRNA levels of *IL-1β* and *IL-10*. Moreover, the expression levels of the TNF-α protein in the guava group were significantly lower than those in the other two groups (Figure 7H). Previous studies confirmed that lycopene-rich extracts exerted significant anti-inflammatory effects, including inhibiting leukocyte migration, stabilizing mast cell membranes, and downregulating the expression of inflammation-related genes. Its underlying mechanism might be related to inhibiting the production of pro-inflammatory cytokines and regulating apoptotic signaling pathways [44,47,48]. Our previous study also found that lycopene could inhibit the activation of the TLR-4/NF-κB signaling pathway by lipopolysaccharides, thereby reducing inflammation in the jejunum of mice and maintaining their intestinal health [7]. In addition to its ability to scavenge ROS, lycopene also possesses anti-inflammatory properties. However, few studies have compared the anti-inflammatory effects of lycopene from different sources. This study indicated that lycopene-rich extract derived from red guava had greater anti-inflammatory potential than lycopene derived from tomatoes. The potential mechanism might be that red guava-derived lycopene-rich extract possessed stronger antioxidant capacity, which could more effectively scavenge excess ROS, thereby inhibiting the activation of the NF-κB pathway and alleviating inflammation. However, further studies are required to clarify the mechanism.

## 4. Conclusions

The aim of this experiment was to extract lycopene from tomatoes, cherry tomatoes, guavas, carrots, and watermelons by using the UAE method with ethyl acetate as the extraction solvent and to comparatively analyze the differences in their antioxidant capacities in vitro and in vivo and their anti-inflammatory capacity in vivo. The results indicated that except for tomatoes, the lycopene concentrations in the other four samples were approximately 30 mg/100 g FW. Analysis of the in vitro antioxidant capacity revealed that the red guava-derived lycopene-rich extract exhibited significantly higher ABTS and DPPH scavenging rates and FRAP concentrations than the other four sources. Additionally, the results of the in vivo studies revealed that, compared to tomato-derived lycopene-rich extract, the red guava-derived lycopene-rich extract significantly enhanced antioxidant enzyme activity and decreased MDA concentrations in mouse serum, liver, and GAS and increased the expression of antioxidant-related genes (*GR*, *GST*, *GPx*, *CAT*, *SOD1*, etc.) in mouse liver and GAS. This process was primarily mediated through the Nrf-2/Keap1 signaling pathway. In addition, mice in the guava-derived lycopene group had lower serum levels of tumor necrosis factor-α (TNF-α) and interleukin-6 (IL-6), suggesting that lycopene derived from red guavas showed greater anti-inflammatory potential. Therefore, lycopene-rich extracts from different sources exhibited variations in content and antioxidant capacity both in vitro and in vivo. Furthermore, the results indicated that the lycopene-rich extract derived from red guava demonstrated superior antioxidant potency in both in vitro and in vivo studies, as well as greater anti-inflammatory potential, holding significant potential value for applications in the field of food science, including practical implications for food processing and functional food development. Based on the results of this study, subsequent research could further investigate the functional effects of pure lycopene compounds from different sources.

Tomatoes are the predominant raw material for industrial lycopene production at present. Meanwhile, fruits and vegetables such as guavas, watermelons, and carrots also have abundant reserves and are highly renewable. By comparing the functional capacities of lycopene from these sources, it could provide a reference for optimizing raw material selection in the food industries.

## Figures and Tables

**Table 1:** All-*trans*, *cis*-isomer and β-carotene contents of extracted samples.

Sample	Total Lyc Content (mg/100 g FW)	All-*trans* Content (mg/100 g FW)	Total *cis* Content (mg/100 g FW)	β-Carotene Content (mg/100 g FW)
Tomato	13.72 ± 0.27 ^c^	13.19 ± 0.25 ^c^	0.53 ± 0.02 ^c^	0.80 ± 0.02 ^d^
Cherry tomato	37.12 ± 0.23 ^a^	36.08 ± 0.20 ^a^	1.04 ± 0.04 ^a^	1.95 ± 0.05 ^b^
Guava	29.09 ± 0.24 ^b^	28.86 ± 0.23 ^b^	0.23 ± 0.01 ^d^	0.48 ± 0.01 ^e^
Carrot	37.35 ± 0.31 ^a^	36.45 ± 0.31 ^a^	0.90 ± 0.01 ^b^	5.78 ± 0.12 ^a^
Watermelon	29.73 ± 0.58 ^b^	29.23 ± 0.57 ^b^	0.50 ± 0.01 ^c^	1.32 ± 0.05 ^c^

Data are expressed as means ± SEM (n = 3 per group). ^a–e^ Columns with different superscript letters indicate significant differences (*p* < 0.05).

## Data Availability

The original contributions presented in this study are included in the article/Supplementary Materials. Further inquiries can be directed to the corresponding author.

## Supplementary Materials

The following supporting information can be downloaded at: https://www.mdpi.com/article/10.3390/foods15101765/s1. Figure S1: Moisture content of samples before extraction; Figure S2: Effects of lycopene on body weight, feed intake, weight of liver and GAS in mice; Table S1: Primer sequences of the target and reference genes.
