# Supplementary material for: Comparative Analysis of the In Vitro and In Vivo Antioxidant and Anti-Inflammatory Capacities of Lycopene Extracts from Different Sources
Source: Foods. 2026 May 17;15(10):1765. doi: 10.3390/foods15101765 (PMC13205308; doi:10.3390/foods15101765)
Supplement: Supplementary file 1 [file foods-15-01765-s001.zip › foods-4246570-supplementary.pdf]

Supplementary data

Supplemental Figure

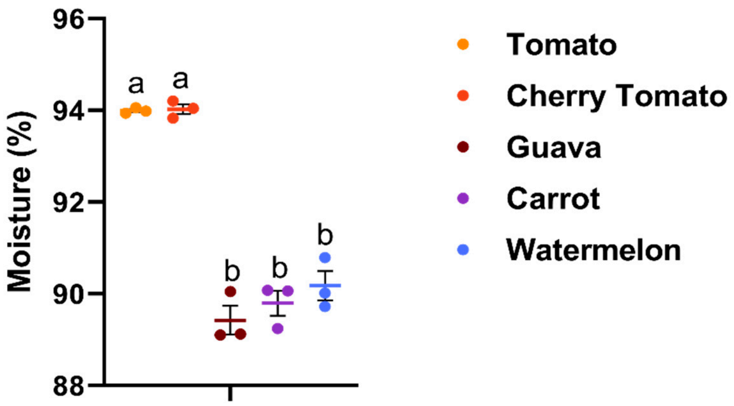

Supplemental Figure S1. Moisture content of samples before extraction. Data were expressed as means ± SEM (n = 3 per group).

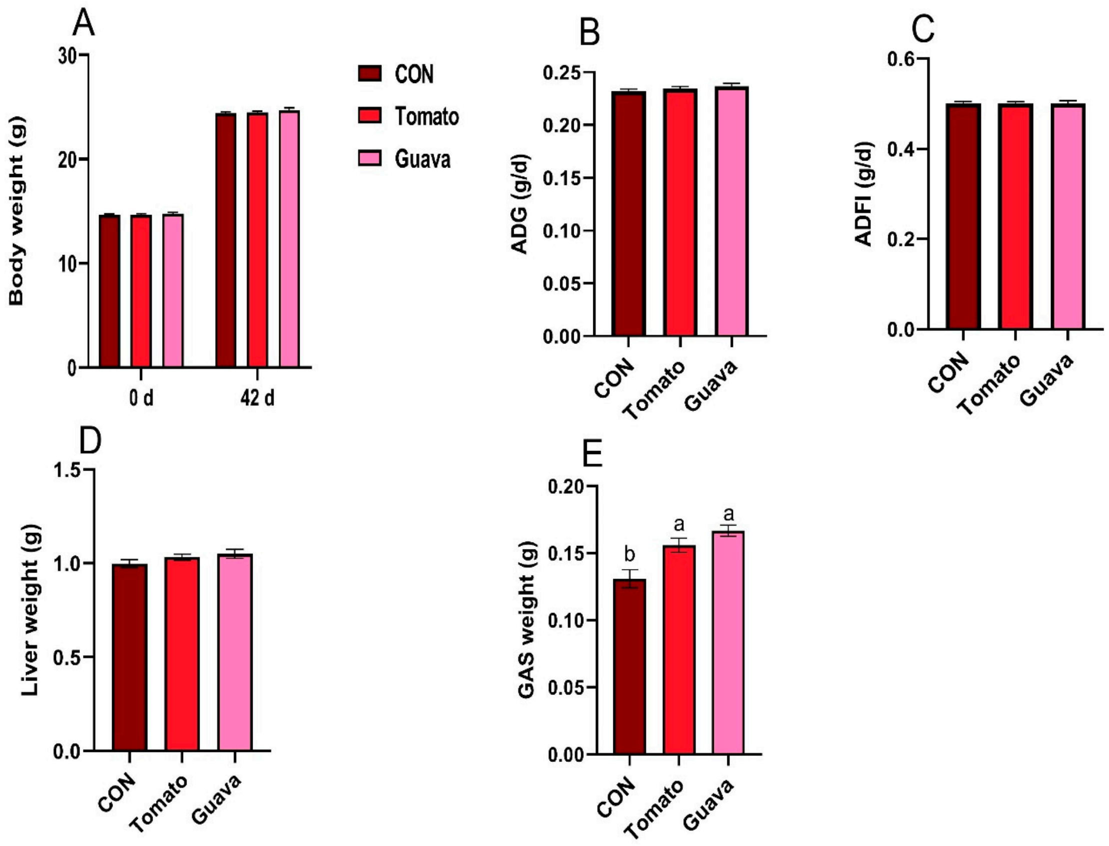

Supplemental Figure S2. Effects of lycopene on body weight, feed intake, weight of liver and GAS in mice. Data were expressed as means ± SEM (n = 12 per group). ADG, average daily gain; ADFI, average daily feed intake; GAS, gastrocnemius muscle.

Supplemental table

Supplemental Table S1. Primer sequences of the target and reference genes

Genes	Primer	Sequence (5'→3')
Nrf-2	Forward	5'-AAAGCACAGCCAGCACATTC-3'
	Reverse	5'-TGGGATTCACGCATAGGAGC-3'
Keap1	Forward	5'-CTCAACCGCTTGCTGTATGC-3'
	Reverse	5'-TTCAACTGGTCCTGCCCATC-3'
NQO1	Forward	5'-AGGATGGGAGGTACTCGAATC-3'
	Reverse	5'-AGGCGTCCTTCCTTATATGCTA-3'
HO-1	Forward	5'-AAGCCGAGAATGCTGAGTTCA-3'
	Reverse	5'-GCCGTGTAGATATGGTACAAGGA-3'
TXNRD2	Forward	5'-TACAGCAATGTTCCCACAACTGTC-3'
	Reverse	5'-CTATCCGCCACCGTGAACTC-3'
TXNRD1	Forward	5'-TACTGCATCAGCAGTGATGATC-3'
	Reverse	5'-CCATGTTCTTCCATGTGTTCAC-3'
GR	Forward	5'-GACACCTCTTCCTTCGACTACC-3'
	Reverse	5'-CCCAGCTTGTGACTCTCCAC-3'
GST	Forward	5'-GGATGGGGACTTCGTCTTGG-3'
	Reverse	5'-TCAGGAGGTACGGGCTGTC-3'
GPx	Forward	5'-CTCAAGTACGTCCGACCTGG-3'
	Reverse	5'-TAAAGAGCGGGTGAGCCTTC-3'
CAT	Forward	5'-GCGGATTCCTGAGAGAGTGG-3'
	Reverse	5'-TGGAGAACCGAACGGCAATA-3'
SOD2	Forward	5'-CAGACCTGCCTTACGACTATGG-3'
	Reverse	5'-CTCGGTGGCGTTGAGATTGTT-3'
SOD1	Forward	5'-GGAACCATCCACTTCGAGCA-3'
	Reverse	5'-CCCATGCTGGCCTTCAGTTA-3'
NF-κB1	Forward	5'-ATGGCAGACGATGATCCCTAC-3'
	Reverse	5'-TGTTGACAGTGGTATTTCTGGTG-3'
TNF-α	Forward	5'-CATCTTCTCAAAATTCGAGTGACAA-3'
	Reverse	5'-TGGGAGTAGACAAGGTACAACCC-3'
IL-1β	Forward	5'-GAAATGCCACCTTTTGACAGTG-3'
	Reverse	5'-TGGATGCTCTCATCAGGACAG-3'
IL-6	Forward	5'-TAGTCCTTCCTACCCCAATTTCC-3'
	Reverse	5'-TTGGTCCTTAGCCACTCCTTC-3'
IL-10	Forward	5'-AGCCTTATCGGAAATGATCCAGT-3'
	Reverse	5'-GGCCTTGTAGACACCTTGGT-3'
GAPDH	Forward	5'-AGGGCATCTTGGGCTACAC-3'
	Reverse	5'-TGGTCCAGGGTTTCTTACTCC-3'

Nrf-2, nuclear erythroid 2-related factor 2; Keap1, Kelch-like ECH-associated protein 1; NQO1，NAD(P)H dehydrogenase quinone 1; HO-1, heme oxygenase 1; TXNRD1/2, thioredoxin reductase 1/2; GR, glutathione reductase; GST, glutathione S-transferase; GPx, glutathione peroxidase; CAT, catalase; SOD1/2, superoxide dismutase 1/2; NF-κB, nuclear factor kappa B; IL-6, interleukin 6;

IL-1β, interleukin 1β; IL-10, interleukin 10; TNF-α, tumor necrosis factor -α; GAPDH, glyceraldehyde-3-phosphate dehydrogenase.
